# Distinguishing the Effect of Time Spent at Home during COVID-19 Pandemic on the Mental Health of Urban and Suburban College Students Using Cell Phone Geolocation

**DOI:** 10.3390/ijerph19127513

**Published:** 2022-06-19

**Authors:** Pelin Ayranci, Cesar Bandera, NhatHai Phan, Ruoming Jin, Dong Li, Deric Kenne

**Affiliations:** 1 Martin Tuckman School of Management, New Jersey Institute of Technology, Newark, NJ 07102, USA; bandera@njit.edu (C.B.); phan@njit.edu (N.P.); 2 Department of Computer Science, Kent State University, Kent, OH 44240, USA; rjin1@kent.edu (R.J.); dli12@kent.edu (D.L.); 3 College of Public Health, Kent State University, Kent, OH 44240, USA; dkenne@kent.edu

**Keywords:** COVID-19, depression, anxiety, mobile health, home stay, GPS, smart phone, PHQ-9, GAD-7

## Abstract

The aim of this study was to assess the correlation of depression and anxiety with time spent at home among students at two universities—one urban and the other suburban—during the COVID-19 pandemic. Methods: Geolocation data from the smartphones of 124 participants were collected between February 2021 and May 2021. The level of depression was estimated by the PHQ-9 and PHQ-2 screening tools, and anxiety scores were estimated by the GAD-2 and GAD-7 screening tools. Results: 51% of participants in the PHQ-9 surveys indicated mild to severe depression. Participants spent on average 75% of their time at home during COVID. Time spent at home had a positive correlation with the mental health of urban students but a negative correlation with suburban students. The relation between the time at home with mental health was stronger among female participants than among male participants. Correlations between female depression, anxiety, and time at home were significant. Conclusions: Lockdown and distance learning contributed to the high levels of depression in university students. This research highlights the importance of time spent at home for mental health being during the pandemic and the importance of distinguishing between urban and suburban settings when formulating public health recommendations. Quality of time spent at home versus time spent outside differentiated the mental well-being of students located in different environments. Staying at home may be recommended for students without access to safe outdoor places as it is associated with lower levels of depression.

## 1. Introduction

The world health organization reports that mental health is one of the leading causes of disability worldwide, and it is a major contributor to the overall burden of disease. The COVID-19 pandemic highlighted the importance of considering mental health as an integral part of public health response [1,2].

Starting in mid-March of 2020, universities had to implement social distancing and remote learning protocols. Students had to limit the time they spent outside or in classrooms, and attend classes from inside their houses, which increased the time they spent at home. The pandemic limited in-person social interactions and increased the amount of time spent on smartphones [3], the latter of which represents an opportunity to monitor the behavioral patterns of individuals [4].

Researchers seek to exploit the ubiquity, embedded sensors, and persistent interaction of smartphones to assess the mental health of a smartphone user [5]. Smartphones record behavior patterns of users with high temporal precision and without the need for user intervention reducing the response rate. Only 59% of the users respond to PHQ surveys [6]. Global Navigation Satellite System (GNSS) data obtained from smartphones are particularly important to identify movement patterns and time spent at specific locations. Analyzing GNSS data instead of asking participants where they spent their time has recently become a popular research method [7,8].

The Patient Health Questionnaire (PHQ) and General Anxiety Disorder (GAD) questionnaires are frequently used in mental well-being screening. The GAD is frequently used to measure the severity of anxiety in patients, whereas the PHQ measures depression severity in general medical and mental health settings.

The PHQ-2 and PHQ-9 are validated survey instruments widely used to measure depression [9,10]. The PHQ questions were developed by Drs. R.L. Spitzer, J.B.W. Williams, and K. Kroenke with Pfizer. Pfizer, Inc. (New York, NY, USA) holds the copyright for the PHQ and has made it publicly available for use by researchers without further permission [9,10]. It contains nine questions, which assess the criteria for depression defined by the Diagnostic and Statistical Manual of Mental Disorders (or *DSM-5*) [11]. We have included a sample PHQ-9 questionnaire in Appendix E. The PHQ-9 questionnaire also assess anhedonia, although demoralized patients often do not experience anhedonia [12].

The aim of this study is to measure the time university students spent at home during COVID-19 unobtrusively and evaluate its correlation with the individual’s levels of depression and anxiety severity as self-reported using PHQ and GAD scores. We use GNSS data to identify the time spent at home, instead of relying on students’ recollection, and automatically label GNSS measurements as “at home” or “away” without student intervention.

Suburban students’ access to outdoor open spaces might not have changed during the pandemic, whereas urban students’ city life (e.g., concerts, theaters, shopping) may have been more curtailed by social distancing protocols. An additional aim of this study was to measure the moderating effect of one’s setting (urban or suburban) on the impact of time away from home on mental health. Public health policy could account for this moderating effect by distinguishing urban and suburban protocols that meet both social distance and mental health requirements.

## 2. Materials and Methods

### 2.1. Population, Study Sample, and Collection of Data

We collected data from 124 university students. More than 50% of the students were from Kent State University (KSU) which is located in a suburban area, and the other half were from the New Jersey Institute of Technology (NJIT) which is located in an urban area. Therefore, we have a balanced sample of urban and suburban students. Students in the study consisted of undergraduate and graduate-level students.

We opted to recruit university students for two reasons. First, university students should have relatively good access to smartphones and related technologies. Second, university students should be more credible and easier to be motivated than other sources (e.g., recruiting test subjects on crowd-sourcing websites). Software start-up Unknot.id developed and deployed the data collection app. The data collection app is the application the students download into their smartphones. We performed two data collection runs, one at NJIT (starting mid-April) and one at KENT (starting mid-May). Each run lasted about three months.

Due to licensing limitations, only the Android version of the app was eventually developed and was distributed privately instead of going through the app market. This limited the scope of the data collection to Android users.

The app has two main components: The frontend (i.e., the app itself on the phone, illustrated in Appendix A), which is the part of the application users interact with. The main functions of the app are: (1) Acquire sensor data from the smartphone; (2) Allow users to label their current/upcoming activities; (3) Detect user activities and remind users to provide labels of their activities; (4) Upload the collected data and labels to the backend. The backend is the portion of the applications people do not see. The backend is responsible for organization and storage of data collected. In addition, users could register their own accounts on the app, turn off the types of data that they do not want to share, and track their upload history. The types of data that the app can collect are listed in Appendix B. The availability field marks how many of our student volunteers can collect each data type. The description of terms used in Section 2 is listed in Appendix F.

### 2.2. Data Analysis Strategies

Sensor data required extensive preprocessing steps. Data processing steps have three objectives: (1) Remove or fix the errors and imperfections in the raw data, including repeated data points, gaps between data points, fluctuating sampling rates, and inaccurate labels. (2) Align the sensor data with the labels across time (walking, driving, sitting), such that each unit of data points is associated with its correct label; and (3) Convert the format of the raw data into short sessions of data points with multiple channels (i.e., accelerometer data has 3 channels, x, y, z, in the data representation) that will serve as basic input data units of the models.

In our preliminary results, subjects were divided into two groups using their responses to the PHQ and GAD questionaries. Thresholds for each measure were used to divide individuals into two groups: (1) likely mild or greater depression or anxiety, or (2) none/normal. For the short survey (PHQ-2, GAD-2), scores ≥ 3 suggest clinical depression or anxiety. For long surveys (PHQ-9, GAD-7), scores ≥ 5 suggest depression or anxiety symptoms that are in the range of at least mild.

Higher PHQ-9 scores indicate decreased functional status and increased symptoms that keep the subjects away from their daily activities.

#### Overview of Datasets Used in Experiments

Once the data are processed by previously described steps, we have clean data. The clean data include the sensor measurements listed in Appendix B. It is important to mention the discrepancies in the datasets we use in experiments. In this section, we will analyze the dataset we have in more detail.

In our dataset, 124 participants completed at least one survey. Not every individual who completed the survey participated in the study long enough for us to consider them as participants because some of the individuals only completed one survey and did not use the software or simply chose to opt out of the study. The study collected 894 surveys from 124 individuals. Two versions of the GAD and PHQ were utilized. The long version included all 7 GAD questions and all 9 PHQ questions. Short versions included only the first 2 questions of each measure. To make the scores from both versions of the measures comparable, we normalized scores.

Experiments consider surveys as a data point and participants as a data point. When a survey is a data point, we have 894 data points, and when participants are a data point, we have 124 data points. Specific questions in the survey indicate depression and anxiety. In the short survey, the first two questions of each tool measure anxiety or depression (e.g., GAD-2, PHQ-2). In the long survey, all 7 questions measure anxiety (GAD-7 questionnaire), and all 9 questions measure depression (PHQ-9). In our sample, the highest anxiety is indicated by 21 points (out of a possible 27).

Scores between 1 and 4 are considered minimal depression in the PHQ-9 and minimal anxiety in the GAD-7, scores between 5 and 9 mild depression in the PHQ-9 and mild anxiety in the GAD-7, 10–14 moderate depression in the PHQ-9 and moderate anxiety in the GAD-7, 15–19 moderately severe depression in the PHQ-9 and moderately severe anxiety in the GAD-7, and 20–27 severe depression in the PHQ-9 and severe anxiety in the GAD-7. Several studies consider scores higher than 5 as depressed for the PHQ-9 and anxious in the GAD-7 [7,13,14]. In the PHQ-2 and GAD-2, the sum of the scores ≥ 3 (average score ≥ 1.5) was recommended by several researchers as cut-off points between the normal range and probable cases of depression or anxiety, respectively [15,16].

### 2.3. Assessing the Level of Depression and Anxiety

The total score on the PHQ and GAD questionaries ranged from 0 (not at all) to 3 (nearly every day) to identify the graded severity of symptoms. In the most severe cases, we expect a severity score of 27, whereas a 0 severity score signals lack of evidence for depression/anxiety symptoms for the PHQ-9 and GAD-7 which are the long form of the surveys [13,14]. In our research, we use both PHQ-9 and PHQ-2 scores as the ground truth to check our predictions for depression. The GAD-7 and GAD-2 are the anxiety detection modules that we use as the ground truth to check anxiety predictions. The threshold criterion for the short survey (GAD-2) is 3. If the survey score is larger than 3, the short survey respondent is showing signs of depression. The long survey respondent with a total score of 5 and above is showing signs of depression. Anxiety modules have the same clinical thresholds.

### 2.4. GNSS Data Analysis

Geolocation data were retrieved from each participant’s cell phone. For a complete GPS data set, we expect to see 24 h of data every day indicating where users spend their time. Appendix D shows the distribution of the daily data of the users. We wanted to derive the ratio of time users spent at home and outside locations. We evaluated the ratio of time spent at home and depression scores in the PHQ-4 survey.

The calculation of time away and at home follows seven steps. Firstly, the labeling of a location as a participant’s home begins with a reduction in geospatial resolution to a grid where the area of a grid cell encompasses the participant’s home (roughly 90 square meters excluding multiple levels). The grid cell most occupied by a participant during the study is subsequently labeled as “home”. We achieve this desired resolution by simply discarding all digits to the right of the third decimal place of the latitude and longitude geospatial measurements (i.e., with no more than one millidegree resolution). One millidegree in latitude represents 111.2 m, and in longitude, one millidegree represents 84.3 m at NJIT and 83.7 m at KSU. Any participant activity within the one square millidegree grid cell (roughly 9340 square meters) encompassing the participant’s home will thus be considered “at home”.

Second, to determine the house location for each user, we used the frequency method. We labeled the most frequent location as home. Third, we used all geolocation data that were sorted chronologically. Fourth, time spent in-home or outside locations was found by the difference between consecutive periods. For example, a user is located at (latitude1, longitude1) at 19:30, and the next data we have for this user is 19:45, and the user is still located at (latitude1, longitude1) which is the home location for this user. The 15 min spent on that day belongs to the time spent at home. Fifth, we do not have 24 h of location data for each individual. We include data point (user) even if it does not have 24 h of data. Finally, we used the clinical thresholds for depression and anxiety to cluster depressed and non-depressed individuals.

We also tried the k-means clustering method instead of clinical thresholds, but clinical thresholds did not differ significantly from clinical thresholds which we described in Section 2.2.

We used the method described due to missing labels. In particular, some participants had missing data points when their phones were closed. This is a common problem when dealing with mobile data collection apps that run in the background, as opposed to geolocation apps that run in the foreground such as navigation apps. Our GPS analysis method overcomes the missing data points problem.

We created an “at home ratio” of users for each day in Table 1. For example, if a user spent all day at home, we have 1 as the at home ratio.

### 2.5. Statistical Methods

We analyzed the distribution of features in our dataset in Table 2. Since our sample size was larger than 50, we used the Kolmogorov–Smirnov test to check the normality of the distribution instead of the Shapiro–Wilk test. It is important to note that both tests had the same conclusion about normality in our case [17,18]. Both tests rejected the null hypothesis that our feature dataset follows a normal distribution. We reported the skewness coefficient values to check the distribution further. The skewness coefficient differed from 0 for the features, and for some features it was below −1 or above 1. Values above 1 and below −1 signal a highly skewed distribution. Both statistics measures signal a non-normal distribution. Therefore, using non-parametric measures of statistical inference is suitable for analysis. We used nonparametric measures of Spearman’s rank correlation coefficient and the Mann–Whitney test for statistical analysis.

## 3. Results

A total of 53% of students were from the suburban setting and 46% from the urban setting. NJIT students consisted of 69% male and 31% female participants. A total of 30% of surveys indicated that participants experienced depressive symptoms over the preceding two weeks from the survey, and 28% experienced anxiety. A total of 17% of students who took the short survey were classified as depressed throughout the study with PHQ-2 scores above 3. A total of 51% of students who took the long survey showed signs of mild to severe depression throughout the study with PHQ-9 scores above 5.

### GNNS Analysis

The GNSS analysis found that the average at-home ratio of university students in our sample was over 0.75. One of the interesting characteristics of our sample is having students from both NJIT and Kent State University campuses. We discovered that the correlation between time spent at home ratios and PHQ-2 depression scores of individuals from these campuses differed. One of the most important differences between those two groups is the direction of the correlation in Table 3. In urban settings, when participants spent more time at home, their responses to questionnaires indicated lower depression scores (r*_S_* = −0.45; *p* = 0.01 **). On the other hand, participants in the suburban setting of Kent State University showed signs of depression when they spent more time at home (r*_S_* = 0.22; *p* = 0.09). The same trend repeated for anxiety measures in Table 4.

We saw a strong correlation between time spent at home and depression along with anxiety scores reported for female students at NJIT when we removed the female students that completed fewer than 3 surveys through the study (r*_S_* = −0.85; *p* = 0.01), (r*_S_* = −1; *p* = 0.00). When the average time they spent at home the last week before the survey increased, they had low depression and anxiety scores in Table 5. On the other hand, male correlations were insignificant.

Students in urban settings spent more time at home compared to students in suburban settings during the lockdown period. The difference between time spent at home was significantly different for suburban and urban participants (H(112) = 1975.0, *p* = 0.00). Anxiety scores of suburban students were higher compared to urban settings. Depression and anxiety scores of urban and suburban settings were not significantly different from each other in Table 6.

## 4. Discussion

The COVID-19 pandemic brought lockdowns and social isolation, increasing the time spent at home. Students whose schools switched from in-person instruction to distance learning often participated from home where there was a stable internet connection, and the time they would normally have spent in classrooms was instead added to the time spent at home. A recent study indicated that 80% percent of the students had increased screen times during COVID-19 [19]. Spending time outside is associated with lower stress levels and positive mental health outcomes for the general population [20,21]. An increase in time spent at home has a toll on mental well-being, especially on students. We found that increased time spent at home was associated with higher PHQ-2 and PHQ-9 scores in university students in a suburban setting. However, time spent at home did not have the same relation with students living in urban settings. Instead, a negative correlation among urban students between time at home and PHQ scores indicates a possible effect of indoor and outdoor environment quality on depression levels.

The above distinction between urban and suburban settings has public health implications. Prior to the COVID-19 pandemic, researchers into the disparity between urban and suburban public health found conflicting results. In their seminal paper, Sturm and Cohen [22] found a disparity in physical health but not mental health. Subsequently, Cumbrera [23] found a disparity in mental health, but proposed it is associated with a disparity in socioeconomic status. The unprecedented social isolation imposed by the pandemic offers researchers the opportunity to evaluate this disparity in a new light. Moreover, the similarity in socioeconomic status among KSU and NJIT students allows us to discount that confounding factor. Social distancing policies can thus include time-at-home recommendations that account for urban/suburban setting while adhering to overall safety protocols.

Although the ratio of time university students spent inside was not investigated, university students were questioned relating to their mental well-being by several researchers. They reported alarming anxiety and depression scores among students during the COVID-19 pandemic [8,24,25]. Our research also showed that 51% of the students showed signs of depression during the COVID-19 lockdown period.

Long-term studies of depression concluded that women are more likely to suffer more from depression than men [26]. Our findings also confirm this trend. Depression scores of female participants were higher than male participants, and the difference is statistically significant. In our sample, males tended to have lower depression scores from PHQ-2 questionnaires. Anxiety scores of female and male participants also indicated that women had higher anxiety scores on average throughout the study. The female average GAD-2 score was 1.47 and the male average GAD-2 score 1.2. Studies conducted during COVID-19 also confirmed these findings [27,28].

During the COVID-19 pandemic, crime rates climbed to higher levels in cities [29]. Economic consequences of the COVID-19 pandemic connected with the global economy affected the cities, and the increased poverty of cities translated into high possible crime rates [29]. The suburban and urban places in our sample followed the same trend. In our sample, the crime index was higher in the urban location than the suburban location according to FBI crime data published in September 2021 [30]. Studies on people living in crime hot spots and crime cold spots indicate that people living in crime hot spots have a 61% higher symptomology score for depression than people living in crime cold spots [31]. We observed this trend in our sample, and our recommendation is if students do not have access to safe outdoor places, staying home will be a better option for their depression symptoms given the higher depression scores of urban residents in relation to the time they spent outside.

## 5. Conclusions

The COVID-19 lockdowns and distanced learning had a toll on the mental well-being of university students. Time at home substituted time normally spent at university campuses with friends. We found that social isolation at home had a negative relation with depression in students living in suburban areas, but a positive relation with depression on students living in urban areas. We highlight the moderating effect of location on the relation between time spent at home and depression levels.

This research highlights the importance of time spent at home for mental health being during the pandemic. Quality of time spent at home versus outside differentiated the mental well-being of students located in different environments. Creating a mindful safe space at home and outside is important for mental health.

A strength of the study comes from a reduction in the reliance on human-reported factors through the use of ubiquitous smartphone technology that does not require user intervention. Behavioral features in our study are retrieved from sensor data as opposed to human-reported survey answers used in previous studies [32]. Moreover, participants in our study were rewarded for their participation. Previous studies found that monetary rewards motivate the subject and yield a higher response rate and response [33]. The weaknesses of the study come from the reliance on human-reported depression and anxiety measures. Although patient health questionnaires are commonly used by researchers, their accuracy still depends on human subjects’ recall. While we consider the PHQ-9 to be a well-validated measure of depression, it is still important to think about factors other than depression that may cause these relationships. We are not aware of the chronic illnesses of the individuals in the dataset that may affect the relationships.

A large-scale study with a more diverse population is needed to confirm the generalizability of our findings. Since this study has a limited number of university students from KSU and NJIT, a diverse population would confirm the generalizability of the findings. Another study weakness is the duration of the study. A longer data collection duration for this study is needed to confirm the results obtained from the study further. Moreover, poor mobile networks affected the quality and coverage of GNSS signals. Frequent mobile phone usage drained the battery and decreased the available sensors for the analysis.

## Figures and Tables

**Table 1:** Summary statement on the time spent at home.

Variable	n	M	SD	min	max
1 Time spent at home	6262	0.76	0.27	0	1

**Table 2:** Summary statement on the distribution of variables.

Variable	Mean	Std.	Skewness	Kurtosis	Median	K-S Test	Sig
1. Time at home Ratio	0.76	0.27	−1.19	0.47	0.87	0.53	* 0.00
2. Depression (PHQ-2)	1.51	1.60	1.02	0.43	1	0.50	* 0.00
3. Depression (PHQ-9)	6.73	5.83	0.78	−0.001	6	0.74	* 0.00
4. Anxiety (GAD-2)	1.55	1.58	1.03	0.58	1	0.50	* 0.00
5. Anxiety (GAD-7)	5.69	5.19	0.95	0.311	5	0.71	* 0.00

* *p* < 0.05, *p* > 0.05.

**Table 3:** Summary statement on the Spearman correlation of participants.

Time Spent at Home	Location			
	Urban *		Suburban	
	PHQ-2 Score (Depression)			
Spearman Correlation	−0.45	(*p* = 0.01)	0.22	(*p* = 0.09)

* more than 3 surveys.

**Table 4:** Summary statement on the Spearman correlation of participants.

Time Spent at Home	Location			
	Urban *		Suburban	
	GAD-2 Score (Anxiety)			
Spearman Correlation	−0.48	(*p* = 0.007)	0.30	(*p* = 0.06)

* more than 3 surveys.

**Table 5:** Summary statement on the Spearman correlation of time at home measure.

Time Spent at Home	Spearman Correlation			
	Depression		Anxiety	
	PHQ-4			
Female at NJIT *	−0.85	(*p* = 0.01)	−1.0	(*p* = 0.00)

* more than 3 surveys.

**Table 6:** Summary statement on the urban-suburban time spent at home, depression, anxiety.

Measure	Urban		Suburban		*p*
	M	Median	M	Median	
Spent at home (All)	0.82	0.89	0.76	0.80	* 0.00
Spent at home (PHQ-4)	1.42	1.35	1.67	1.29	0.90
Anxiety (GAD-2)	1.32	1.2	1.85	1.5	0.15

* *p*-value was calculated using the Mann–Whitney test.

**Figure A1:**
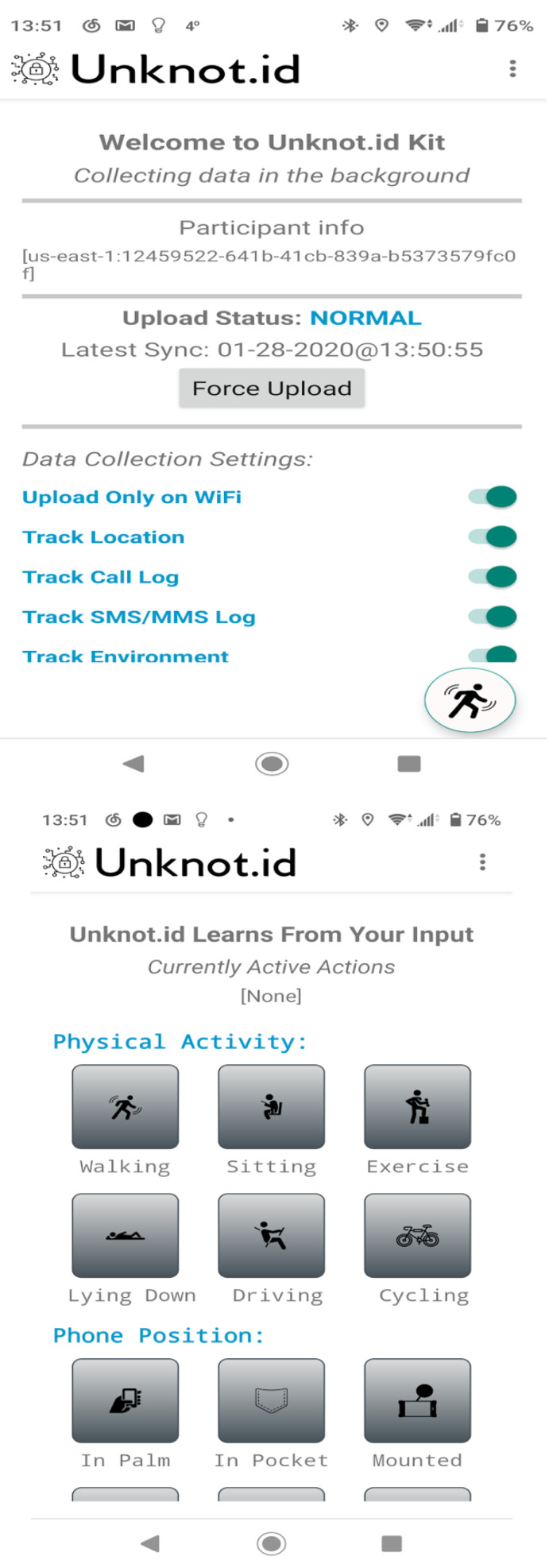
Application Home Page.

**Figure A2:**
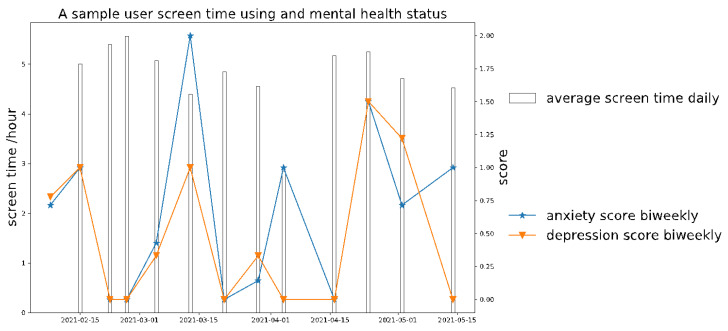
Sample User Mental Health Status Change.

**Figure A3:**
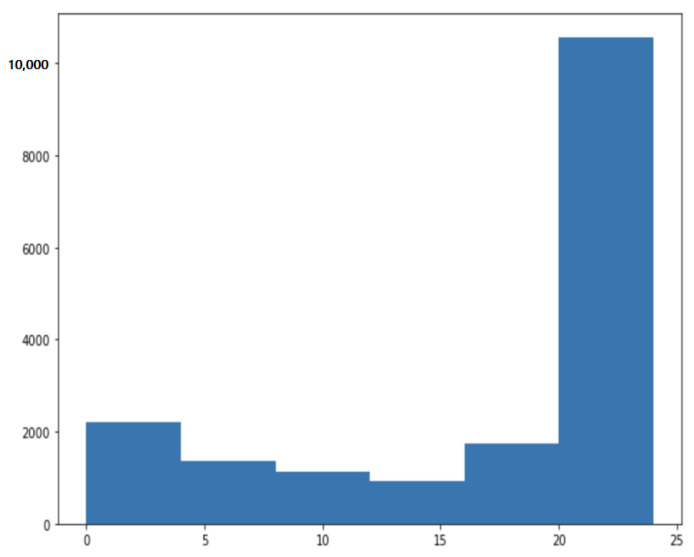
Daily GPS data distribution of individuals (24 h).

**Figure A4:**
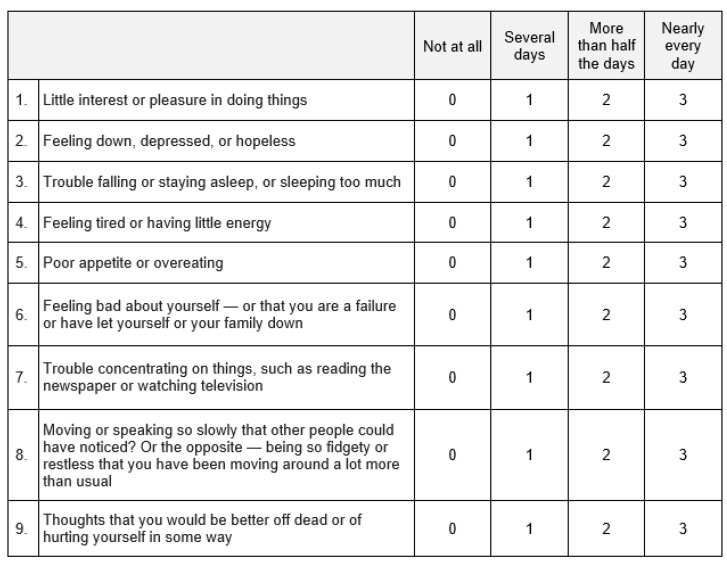
PHQ-9 Questionnaire.

## Data Availability

We acknowledge all the students who participated in this study to make this study possible.

## Appendix B

**Table:**

Sensor	Type	Sampling Rate	Availability	Description
Accelerometer	Motion sensor	5 Hz~50 Hz	114/116	Measures the linear acceleration (including gravity)
Gyroscope	Motion sensor	5 Hz	114/116	Measures the angular speed
Rotation	Motion sensor	5 Hz	114/116	Provides the rotation vector component
GPS	Position sensor	Variable	110/116	Typical GPS data
Magnetometer	Position sensor	5 Hz	113/116	Measures the geomagnetic field strength
Pressure	Environment sensor	5 Hz	83/116	Measures ambient air pressure
Light	Environment sensor	Variable	110/116	Measures ambient light level (illumination)
Battery log	Phone state sensor	Variable	116/116	Measures the smartphone’s battery level
Proximity	Phone state sensor	Variable	109/116	Measures the distance between the screen and an object
Power state	Phone state sensor	Variable	115/116	Different values indicating different state/event of the smartphone
Call log	User data		98/116	Logs the calls that the user had
SMS log	User data		98/116	Logs the short messages (SMS) that the user had

The sampling rate is estimated from observation; actual values vary. The availability means that, out of the 116 participants, how many of them provided this data type.

## Appendix F

**Table:**

Term	Explanation
PHQ	Patient health questionnaire
GNSS	Global navigation satellite system
Crowdsourcing	Obtaining work from large group of people via internet
Deployment	Getting an application to work in a target device
Data collection app	Final application product
Frontend	The part of the applications users interact with
Backend	Backend is responsible for organization and storage of data collected
Geographic grid	Grid defines the latitude and longitude positions
K-means	Unsupervised machine learning method to cluster similar items into the same groups
